# Investigation of Suitable, Readily Available, Sources of Sulfate‐Reducing Bacteria Inoculum, and Evaluation of Sulfate Reduction Rates Achieved at Different pHs

**DOI:** 10.1111/1758-2229.70081

**Published:** 2025-03-12

**Authors:** Janet Smith, Craig Sheridan, Lizelle van Dyk, Kevin G. Harding

**Affiliations:** ^1^ School of Chemical and Metallurgical Engineering University of the Witwatersrand Johannesburg South Africa; ^2^ Centre in Water Research and Development University of the Witwatersrand Johannesburg South Africa; ^3^ School of Geography, Archaeology and Environmental Studies University of the Witwatersrand Johannesburg South Africa

**Keywords:** acid mine drainage (AMD), circular economy, dissimilatory sulfate reduction (DSR), remediation processes, sustainability

## Abstract

This study investigated the suitability of readily available and naturally occurring sources of microorganisms (inoculum) to use for the cultivation of sulphate‐reducing bacteria (SRB) for acid mine drainage (AMD) remediation. The selected inocula included AMD water (AMD), mud (MUD) and reed‐bed mud (RM) from the AMD surrounds, mealworms (MW), cow dung (CD) and raw sewage sludge (RS). The suitability of the different inoculum sources was evaluated by comparing the SO_4_^2−^ reduction and sulfide (S^2−^) production rates at three different pHs. Experimental results showed that the AMD, MW, MUD and CD inoculum did not produce appreciable reduction of SO_4_^2−^ to S^2−^ and were unsuitable sources of SRB inoculum. The inoculum evaluated in pH 2 media did not achieve SO_4_^2−^ reduction. Of the inoculum assessed in pH 4 media, only the RM inoculum achieved SO_4_^2−^ reduction (40%) with S^2−^ production (36 mg/L). In contrast, a notable S^2−^ production, RS (114 mg/L) and RM (99 mg/L), accompanied the SO_4_^2−^ reduction achieved in the pH 7.5 RS (44%) and RM (30%) samples. The improved S^2−^_produced_/SO_4_^2−^_removed_ conversion ratios for samples pH 7.5 RS (0.14) and pH 7.5 RM (0.17) are indicative of increased SRB activity and the suitability of these inoculum as SRB sources.

## Graphical abstract

Selected sources of naturally occurring microbial communities were added to enrichment media to assess their ability to support the growth and activity of sulfate‐reducing bacteria at different pHs. Results showed that only two of the six sources evaluated; raw sewage (RS), and reed‐bed mud (RM), achieved appreciable reduction of sulfate to sulfide at neutral pH.

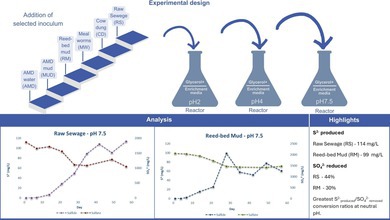

## 1 Introduction

AMD is a highly mineralised, acidic water that forms from the exposure of sulfidic rock to oxygen and water during industrial and mining activities. AMD is a global problem and has caused substantial environmental issues, including the degradation of watercourses and surrounding ecosystems (McCarthy 2011; Simate and Ndlovu 2014). Once AMD formation exceeds natural dilution capacity, remediation is necessary to prevent further ecological damage from this contaminated water. Active remediation processes are effective but often rely on the addition of chemicals, require high capital investment and operating costs and produce large quantities of waste sludge that requires disposal (Simate and Ndlovu 2014; Costa, Verola, et al. 2020; Zhang et al. 2023). Development of a remediation process that uses waste, and/or naturally occurring, renewable materials is vital to ensuring a transition to a circular economy approach that is cost‐effective, sustainable, and uses waste products to produce clean water.

A key biological AMD remediation process is the reduction of SO_4_^2−^ to S^2−^ using SRB via a dissimilatory sulfate reduction (DSR) process. This reaction consumes protons (H^+^) producing S^2−^ and alkalinity, resulting in an increase in pH and the concomitant precipitation of metals out of solution as metal sulfides (Gazea et al. 1996; Greben et al. 2007; Sánchez‐Andrea et al. 2014; Ramla and Sheridan 2015; Nogueira et al. 2021; Ilin et al. 2022). The theoretical S^2−^ production from SO_4_^2−^ reduction is 0.33 (Greben et al. 2007; Gil‐Garcia et al. 2023). In the DSR process, the SRB obtain energy for growth and development by anaerobic respiration using SO_4_^2−^ as an electron acceptor (reduction) and organic carbon or hydrogen as an electron source (oxidation). In this instance, glycerol (C_3_H_8_O_3_) was selected as a non‐ionic carbon source owing to its low toxicity to the environment, easy availability, low cost, and the ability of non‐ionic substrates to allow SO_4_^2−^ reduction at pH 4 (Sánchez‐Andrea et al. 2013). Glycerol has an anticipated high‐energy biomass production owing to its high negative Gibbs free energy change (Sánchez‐Andrea et al. 2013; Ilin et al. 2022; Bagheri Novair et al. 2024). The carbon source is oxidised, and SO_4_^2−^ is reduced as H^+^ ions are consumed, resulting in an increase in pH (Zagury et al. 2006; Bekmezci et al. 2011; Ilin et al. 2022) (Equation 1).

(1) $$7{\mathrm{SO}}_{4}^{2-}+4\;C_{3}H_{8}O_{3}+14H^{+}\;\overset{\mathrm{SRB}}{\to }7H_{2}S+12CO_{2}+16H_{2}O$$

The soluble sulfides (H_2_S) that are formed during the DSR process react with the metals present in the AMD and precipitate out as metal sulfides (Me^2+^) representing the cationic metal—Equation (2) (Zagury et al. 2006; Bekmezci et al. 2011; Ayala‐Parra et al. 2016; Ilin et al. 2022).

(2) $$Me^{2+}+H_{2}S\to \mathrm{MeS}↓+2H^{+}$$

Microorganisms play an essential role in the DSR process, which is influenced by factors such as inoculum, pH, substrate and reactor design (Sánchez‐Andrea et al. 2014). SRB are single‐celled microorganisms that are critical to the DSR process (Ferraz et al. 2021). They occur naturally in different ecosystems and in the gut and oral cavities of humans and animals (Kushkevych, Dordević, et al. 2021; Kushkevych, Kovářová, et al. 2021). Previous studies were able to detect SRB in mine tailings; however, iron and sulfur‐oxidising acidophiles were found to be the most common bacteria found in AMD environments (Johnson and Hallberg 2003; Mendez‐Garcia et al. 2015). Sánchez‐Andrea et al. (2013) reported success in achieving stable SRB enrichments from sediment samples at pH 4 with glycerol. Growth and enrichment of these bacteria are necessary to ensure the preservation of a viable, sustainable SRB colony. Cultivation of SRB is dependent on seasonal (temperature) and other physico‐chemical variables (Sánchez‐Andrea et al. 2014; Kushkevych, Dordević, et al. 2021; Kushkevych, Kovářová, et al. 2021). Physiochemical variables include SRB type, pH, temperature, oxidation–reduction potential (ORP), hydraulic retention time (HRT), electron donor source, chemical oxygen demand (COD)/SO_4_^2−^ ratio, carbon (C)/nitrogen (N) ratio, heavy metal(oid) ions and competing bacterial colonies (Ferraz et al. 2021; Zhang et al. 2022). Previous studies concluded that optimal conditions for the reduction of SO_4_^2−^ by SRB included a completely anaerobic system, pHs between 6.5 and 8.0, and temperatures between 20°C and 35°C (Eloff et al. 2003; Costa, Verola, et al. 2020). Previous work on this topic describes AMD characteristics and the effects of seasonal changes on AMD composition (Smith et al. 2021, 2022). The treatment of AMD using SRB presents an alternate, efficient, cost‐effective and low waste‐generating remediation technology (Costa, De Castro, et al. 2020). This experimental work seeks to further explore a biological process using SRB to remediate AMD waters by investigating potential naturally occurring, easily accessible and sustainable sources of SRB inoculum, and to evaluate the SO_4_^2−^ reduction rates achieved at different pHs using these inoculum sources.

## 2 Materials and Methods

Potential inoculum sources that were collected included: AMD water sampled from a coal mining operation in Mpumalanga, South Africa; mud sampled from an area close to the Mpumalanga AMD source; mud sampled from surrounding reed beds; mealworms (MW) purchased from a pet shop; cow dung (CD) collected from a cattle grazing field in the south of Johannesburg; and raw sewage sludge (RS) that was sampled from the Bushkoppies wastewater treatment facility near Soweto. The impact of pH on SO_4_^2−^ reduction rates of the various SRB inocula was evaluated by conducting this experimental work at three different pHs: pH 2, pH 4 and pH 7.5.

### 2.1 Reactor Set‐Up

**FIGURE 1:**
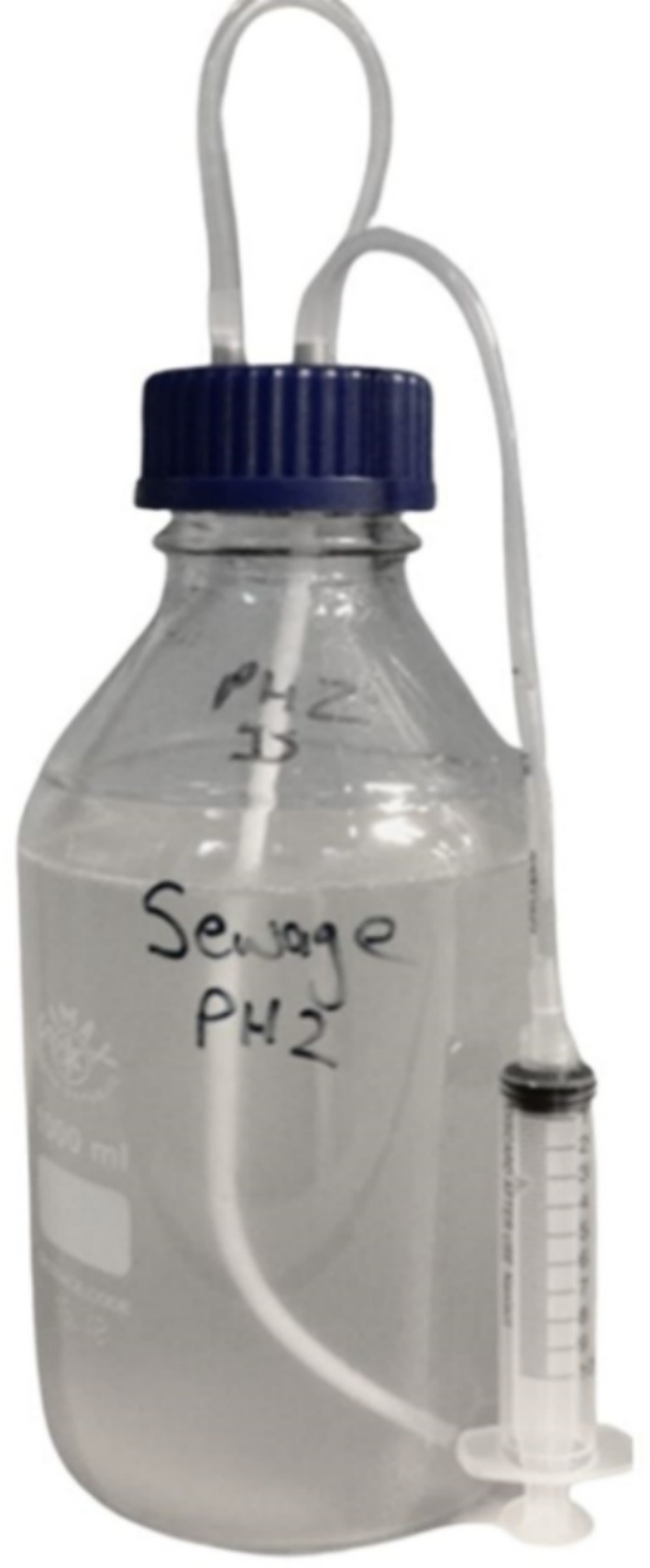
Reactor arrangement for experimental work.

Reactors were assembled using 1000 mL Schott bottles with modified screw‐top fittings, as shown in Figure 1. The tubing allowed access to the sealed reactors for nitrogen sparging and sampling while maintaining a closed anaerobic system. To prevent biological contamination, all reactor bottles and attachments were autoclaved prior to use.

A total of 21 Schott bottles were filled to the 700 mL mark with deionised water (DI) and Merck, analytical grade, reagents were added to each reactor to create a synthetic sulfate‐rich enrichment media with a final sulfate concentration of 2000 mg/L. The growth‐media reagents included: potassium hydrogen phosphate (KH_2_PO_4_), 0.5 g/L (Merck); ammonium chloride (NH_4_Cl) 1 g/L (Merck); yeast extract powder, 1 g/L (Biolab Merck); sodium sulfate (Na_2_SO_4_), 2.9 g/L (Merck) (Greben et al. 2000). In this instance, a 1.15 g/L concentration of glycerol (Merck) was used as the carbon and energy source. The quantity of glycerol added was calculated using the theoretical COD value per unit mass of glycerol (≈1.22 g COD/g glycerol) using reaction stoichiometry to give a final concentration of 1400 mg/L COD (van Haandel and van der Lubbe 2012). This would provide a 0.70 COD/SO_4_^2−^ ratio, which is marginally higher than the theoretical 0.67 g of COD required for SRB to reduce 1 g of SO_4_^2−^ to hydrogen sulfide (H_2_S) (Greben et al. 2000; Greben et al. 2004; Liu et al. 2017; Ferraz et al. 2021). The reactors were divided into three sets of seven reactors. The enrichment media of the first set of reactors was adjusted to pH 2, the second set to pH 4, and the third set to pH 7.5, using 37% hydrochloric acid and/or 1 M sodium hydroxide. Sufficient DI was prepared in separate containers at each of the required pHs for dilution of final reactor volumes to 1000 mL. The reactors and DI solution containers were loosely stoppered and autoclaved at 121°C for 20 min to inhibit biological contamination.

After cooling, nitrogen gas was bubbled through each of the reactors and the pH‐adjusted DI water containers for 10 min to remove oxygen from the solutions. Each reactor set comprised a reactor blank and six reactor samples, each containing a different inoculum source. Table 1 shows the experimental set‐up with the selected inoculum sources and quantity added to each reactor. The blank sample was prepared by replacing the inoculum with 200 mL of DI.

**TABLE 1:** Reactor conditions.

Inoculum number	Inoculum source	Inoculum volume (mL)/mass (g) at pH 2, 4 and 7.5
1	Blank—deionised water (Blk)	200 mL
2	Coal mine AMD (AMD)	200 mL
3	Raw sewage sludge (RS)	200 mL
4	Mealworms (MW)	3 g
5	Cow dung (CD)	5 g
6	Coal mine AMD mud (MUD)	5 g
7	Coal mine AMD reed‐bed mud (RM)	5 g

After the addition of the inoculum, the reactors were made up to a final volume of 1000 mL using the pH‐adjusted DI water, and the reactor lids were tightly fastened. To create an anaerobic environment, nitrogen gas was bubbled through the modified tubing ports for 5 min. Immediately after nitrogen sparging, syringes were attached to the end of the reactor port tubing for drawing off sample aliquots. Reactors were kept at a constant 28°C ± 0.5°C temperature in a Labcon shaking incubator and agitated at 100 RPM.

### 2.2 Parameters Evaluated

The parameters measured included pH, COD, S^2−^ and SO_4_^2−^. A Spectroquant Pharo 300 spectrophotometer was used for the colourimetric determination of S^2−^, SO_4_^2‐^ and COD at wavelengths of 670, 880 and 610 nm respectively. COD was determined after digestion. pH was determined using an Ohaus ST3‐combined pH and mV meter using an ST310 pH electrode with an integrated temperature probe. All parameters were measured at the start of the experiment; pH and S^2−^ were measured on the fourth day and seventh day and then weekly for the duration of the experiment. To limit analysis costs, COD and SO_4_^2−^ analyses were performed less frequently. Test work was terminated for samples when no notable changes in parameter concentrations were observed.

## 3 Results and Discussion

The coal mine AMD, AMD mud, reed‐bed mud (RM), RS and cow‐dung inocula were collected as single grab samples and immediately transported to the biochemistry laboratory located in the Chemical and Metallurgical Engineering building at the University of the Witwatersrand, Johannesburg. The samples were stored at 4°C in a temperature‐controlled refrigerator under anaerobic conditions until needed.

### 3.1 Results

**FIGURE 2:**
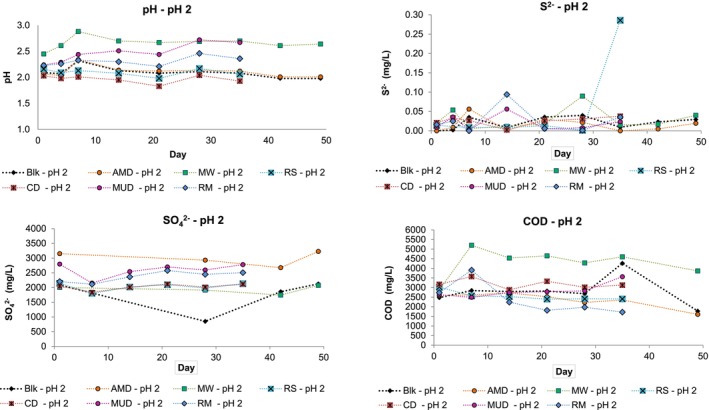
Experimental data for test work performed at pH 2 showing the changes in pH, S^2−^, SO_4_^2−^ and COD concentrations over time.

**FIGURE 3:**
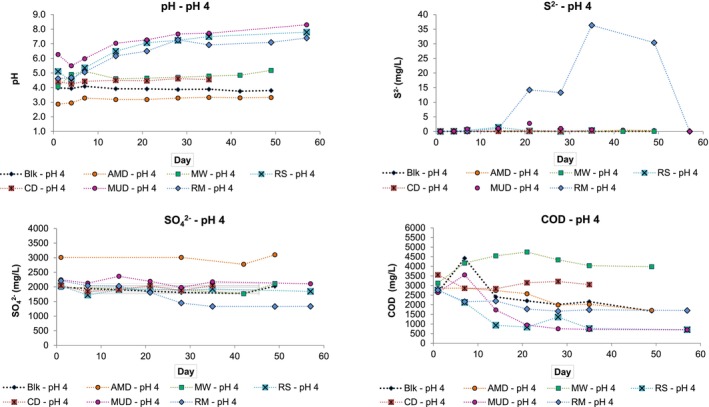
Experimental data for test work performed at pH 4 showing the change in pH, S^2−^, SO_4_^2−^ and COD concentrations over time.

All test work results are presented in Tables S1–S3 and graphical presentation of results for test work performed at pH 2, pH 4 and pH 7.5 are shown in Figures 1, 2, 3. Quality control measures included the analysis of a blank samples containing only enrichment media, which were adjusted to each of the three pHs tested. Method quality control samples were prepared from alternate reagent sources and analysed with each calibration. The control sample concentrations, with averages and percentage error, are included in Tables S4–S7. Replicate sulfate analyses were performed for some samples, and the averages and standard deviations are reported in Table S8.

#### 3.1.1 Testwork Performed at pH 2

Figure 2 shows the results of test work performed at pH 2.

The pH values for the pH 2 samples showed variation over the course of the experiment, but the general trend shown for the Blk, AMD, RS and CD samples was to decrease in pH, reporting marginally lower (more acidic) final pH values. The MUD sample, RM and MW samples increased in pH and reported higher final values.

The RM and MW samples reported S^2−^ concentrations of ≈ 0.1 mg/L on Days 14 and 25 respectively, and the RS sample reported ≈0.3 mg/L on Day 35. S^2−^ concentrations remained low and ranged between 0 and 0.05 mg/L for most samples.

On Day 28, an anomalous decrease in SO_4_^2−^ concentration occurred in the blank sample. Given that there was no concomitant change in other measured parameters and also that SO_4_^2−^ concentrations increased significantly for the next sampling interval, the assumption is that this deviation resulted from analytical error. Most samples reported increased SO_4_^2−^ concentrations at the end of the experiment.

The COD concentration of the MW sample almost doubled on the seventh day, indicating that, as expected, the MW made a significant organic contribution to the reactor media. The MW sample COD concentration then steadily reduced but still reported a final concentration of ≈1000 mg/L more than the initial COD concentration. The MUD sample also reported an ≈1000 mg/L increase in COD concentration at the end of the experimental period. The CD sample reported a 45 mg/L reduction in COD concentration, and the remaining samples reported between 500 and 1000 mg/L final reductions in COD concentration, which was similar to the reduction observed in the blank sample. The decrease in pH and COD observed could be attributed to various chemical interactions between the inorganic and organic components present in the sulfate‐enrichment media.

#### 3.1.2 Testwork Performed at pH 4

Figure 3 shows the results of test work performed at pH 4.

Minor variations in pH occurred in the pH 4 blank sample for the duration of the experiment, reporting lower final pH values on Day 49. The addition of the AMD inoculum to the AMD pH 4 sample decreased the initial pH to 2.8. The pH recovered marginally and reported a final value of 3.32. The same initial decrease in pH did not occur when adding the AMD inoculum to the pH 2 sample, indicating that the pH of the added AMD inoculum was around pH 2. The CD sample achieved a marginal overall increase in pH, while the MW sample pH increased by ≈1 pH unit, which could be attributed to the efficiency of the chitin component present in meal worms in removing acidity and producing alkalinity (Zhang et al. 2022). The addition of RM, RS and MUD inoculum to the pH 4 medium resulted in increased starting pHs of 4.6, 5.1 and 6.3 respectively, with these inocula achieving a significant increase in final pH values over the duration of the testwork. The RM sample increased by 2.8 pH units, RS by 2.7 pH units and MUD by 2.0 pH units. The increased starting pHs for these inocula could be attributed to nutrients present in these inocula resulting in increased neutralising capacity (Merry and Sabljic 2009).

The pH 4 AMD and MW samples achieved the highest S^2−^ concentrations of ≈0.5 and ≈0.4 mg/L respectively, while the pH 4 RS sample achieved its highest S^2−^ concentration of 1.4 mg/L. The pH 4 MUD inoculum sample showed a more pronounced increase in S^2−^ concentration, reporting 0.86 mg/L on Day 7, 1.0 mg/L on Day 14 and 2.8 mg/L on Day 21, after which S^2−^ concentrations decreased. The most notable increase in S^2−^ concentration occurred in the pH 4 RM inoculum sample, increasing from 1.0 mg/L on Day 14 to 36 mg/L on Day 35. Elevated S^2−^ concentrations remained until Day 49, when concentrations declined to 30 mg/L.

The most significant decrease in SO_4_^2−^ concentration for the pH 4 samples occurred in the RM inoculum sample (≈880 mg/L). The RS and MUD inoculum samples achieved decreases in SO_4_^2−^ concentration of between ≈100 and 300 mg/L, which remained for the duration of the experiment. The remaining samples reported varying SO_4_^2−^ concentrations and reported marginally increased SO_4_^2−^ concentrations at the conclusion of the experiment.

The pH 4 MW inoculum sample reported an overall increase in COD concentration. The pH 4 RS and MUD inoculum samples reported an overall decrease in COD concentration of ≈2000 mg/L. The remaining pH 4 samples and blank showed a decrease of between ≈500 and 1000 mg/L in COD concentration during the test work period. The increased COD reduction observed in the RS and MUD inoculum samples could be attributed to an increased utilisation of the available carbon source either from increased SRB activity or from other competing microorganisms, such as fermenting bacteria that might be present in the matrix (Zhang et al. 2022).

#### 3.1.3 Testwork Performed at pH 7.5

**FIGURE 4:**
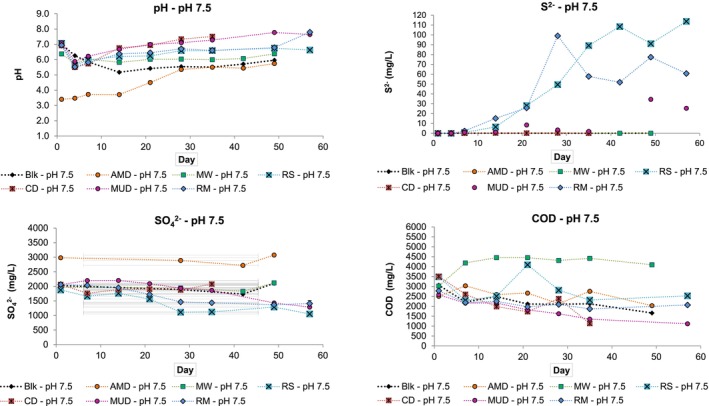
Experimental data for test work performed at pH 7.5 showing the change in pH, S^2^, SO_4_^2−^ and COD concentrations over time.

Figure 4 shows the results of test work performed at pH 7.5.

The addition of the AMD inoculum to the growth media provided sufficient acidity to decrease the starting pH of the pH 7.5 AMD sample to pH 3.5. Despite the lower initial pH, this sample achieved an increase of over 2 pH units over the duration of the experiment. The decreased starting pH (pH 6.4) observed for the MW pH 7.5 sample would indicate that the MW had introduced acidity into the system. The same decrease in pH did not occur in the pH 2 and pH 4 MW samples. Except for the AMD sample, all samples reported a decreased pH on Day 4, followed by an increase in pH. The pH of the pH 7.5 blank sample decreased until Day 14 and then increased to report a final pH of 6.

The pH 7.5 blank sample reported the highest S^2−^ concentration of all the blank samples; however, these concentrations were negligible (< 0.06 mg/L). While small variations in S^2−^ concentration occurred in some samples, the pH 7.5 RS, RM and MUD samples achieved more notable increases in S^2−^ concentration, reporting maximum concentrations of 113, 99 and 36 mg/L, respectively. The pH 7.5 RS, MUD and RM inoculum samples achieved the largest decrease in SO_4_^2−^ concentration, decreasing by 822, 770 and 644 mg/L, respectively.

The COD concentration of the pH 7.5 MW inoculum sample increased by over 1000 mg/L between the first and seventh day, and these elevated COD concentrations remained for the duration of the experiment. The general trend of the remaining samples was to decrease in COD concentration. The largest decrease from the initial COD concentration recorded was for the pH 7.5 CD (2380 mg/L) and pH 7.5 MUD (1465 mg/L) samples.

### 3.2 Discussion

The aim of this test work was to investigate the suitability of six types of readily available and naturally occurring sources of inoculum for the enrichment of SRB. The subsequent activity of the SRB in promoting DSR after addition to a synthetic sulfate‐rich reaction medium was evaluated by assessing the changes in pH, SO_4_^2−^, S^2−^ and COD over time. The inoculum was also evaluated for its capacity for direct use at a range of different pHs. Deviations of the starting pH from the expected values could be due to the addition of the inoculum after the initial pH adjustment of the enrichment media.

Of the inoculum evaluated, the pH 7.5 RS, MUD and RM inoculum achieved a noticeable decrease in SO_4_^2−^ concentration of 44%, 37% and 30% respectively, accompanied by S^2−^ production and COD reduction. The maximum decrease in COD concentration observed in these samples was: pH 7.5 RS (34%), pH 7.5 MUD (57%) and pH 7.5 RM (33%). The reduction of COD could be a result of the oxidation of glycerol by SRB, but could also be attributed to other processes from competing bacteria that might be present in the matrix (Zhang et al. 2022). The pH 4 RM inoculum sample presented a larger percentage decrease in SO_4_^2−^ (40%) and COD (38%) concentration than the pH 7.5 RM inoculum sample, but with lower S^2−^ production (36 mg/L). The decreased S^2−^ production in the pH 4 sample could be a result of the increased hydrogen ions present in a system at low pH, forcing SRB to use up valuable energy to maintain cellular pH homeostasis and causing inhibition of the metabolic activity of SRB, thereby resulting in lower S^2−^ production. Lower pHs could also have enabled the success of other competing bacteria for the carbon sources (Kaksonen and Puhakka 2007; Zhang et al. 2022).

Calculation of the ratio of S^2−^ production to SO_4_^2−^ reduction (S^2−^_produced_/SO_4_^2−^_removed_) showed that the pH 2, pH 4 and pH 7 AMD, MW, MUD and CD inoculum did not achieve appreciable conversion ratios. The reduced conversion ratios for AMD are in line with the anticipated reduced presence of SRB in AMD environments (Johnson and Hallberg 2003; Mendez‐Garcia et al. 2015). Inoculum samples able to realise notable conversion ratios included the pH 7.5 RS inoculum, which achieved the highest conversion ratio of 0.14 on Day 49, and the pH 7.5 RM inoculum, which achieved the highest conversion ratio of 0.17 on Day 28. These inocula demonstrated notable conversion ratios from Day 7 onwards. Conversion ratios reporting lower than the theoretical sulfide production from sulfate reduction ratio of 0.33 could be attributed to the reduction of SO_4_^2−^ to sulfite, thiosulfate and sulfite, or from loss of gaseous H_2_S from reactor fittings or during sampling (Greben et al. 2007; Zhang et al. 2022). An increase in pH and a decrease in COD concentration accompanied the S^2−^ production and SO_4_^2−^ reduction observed for the pH 7.5 RS and RM inoculum samples.

The success of the pH 7.5 RS inoculum in SO_4_^2−^ to S^2−^ conversion could be attributed to the presence of additional and readily available sources of nitrogen, phosphorus, and trace elements in the RS inoculum that would enhance SRB growth (Kaksonen and Puhakka 2007). Similarly, for the RM inoculum, contributions from additional macro and micronutrients and organic matter present in the soil and from the lignocellulosic ruminants would provide additional carbon, nitrogen and phosphorus nutrients. The increased nutrient contribution would provide increased COD/SO_4_^2−^ and carbon‐to‐nitrogen (C/N) ratios in the matrix, resulting in enhanced growth and metabolism of SRB (Zhang et al. 2022). The success of the RM inoculum is in agreement with the observed abundance of SRB in river sediments and soil amendments (Johnson and Hallberg 2003; Sánchez‐Andrea et al. 2013; Ramla and Sheridan 2015).

Data showed a more notable decrease in COD concentration for the pH 4 and pH 7.5 MUD inoculum compared with the pH 4 and pH 7.5 RM inoculum. Of the inoculum tested at pH 4, the pH 4 RM inoculum reported a significant increase in S^2−^ concentrations (36 mg/L), with a maximum SO_4_^2−^/S^2−^ conversion ratio of only 0.041, while the pH 4 MUD inoculum achieved a maximum S^2−^ concentration of ≈3 mg/L. The reduced S^2−^ production in the pH 4 MUD sample could be attributed to the inhibitory effects of low pH on SRB combined with the absence of lignocellulosic material, and thus reduced nutrient content available in the MUD sample (Zhang et al. 2022).

## 4 Conclusions

This work sought to assess the suitability of different, readily available and naturally occurring SRB sources for use in a biologically mediated DSR process for the remediation of AMD. The study evaluated the effectiveness of the SRB cultures to function in different pH environments. The results show that the conversion of SO_4_^2−^ to S^2−^, indicative of the dissimilatory reduction process, did not occur for the AMD water (AMD), mealworms (MW), AMD mud (MUD) and cow dung (CD) inoculum, rendering these unsuitable as natural sources of inoculum for sulfate‐reducing bacteria (SRB) cultivation. The raw sewage sludge (RS) and reed‐bed mud (RM) were able to achieve notable SO_4_^2−^ reduction and S^2−^ production in pH 7.5 media. These inocula achieved the greatest S^2‐^_produced_/SO_4_^2−^_removed_ conversion ratios of all the inocula tested, thus supporting their potential use in biologically mediated DSR remediation processes. This work is important as it contributes to knowledge in the area of sustainable AMD treatment. Using readily available and/or naturally occurring SRB cultures to enhance SRB growth and activity supports the transition to a circular economy model where waste products such as RS is used as a resource in the design of more cost‐effective and sustainable biological AMD remediation processes to produce clean water. Further investigation is needed to establish optimal reactor conditions to achieve the best S^2−^/SO_4_^2−^ conversion rates and to limit potential losses of S^2−^. This work should include the evaluation of using (1) alternate carbon/electron sources that are more efficient than glycerol, (2) different COD/SO_4_^2−^ ratios and (3) varying acclimatisation times for SRB at differing pHs.

## Author Contributions

**Janet Smith:** investigation, writing – original draft, methodology, visualization, formal analysis. **Craig Sheridan:** conceptualization, funding acquisition, writing – review and editing, project administration, supervision. **Lizelle van Dyk:** writing – review and editing, supervision. **Kevin G. Harding:** supervision, writing – review and editing.

## Conflicts of Interest

The authors declare no conflicts of interest.

## Supporting information


Table S1.
Table S2.
Table S3.
Table S4.
Table S5.
Table S6.
Table S7.
Table S8.

## Data Availability

The data that supports the findings of this study are available in the Supporting Information of this article.
