# Supplementary material for: Investigation of Suitable, Readily Available, Sources of Sulfate‐Reducing Bacteria Inoculum, and Evaluation of Sulfate Reduction Rates Achieved at Different pHs
Source: Environ Microbiol Rep. 2025 Mar 12;17(2):e70081. doi: 10.1111/1758-2229.70081 (PMC11897903; doi:10.1111/1758-2229.70081)
Supplement: Supplementary file 1 — Table S1. Table S2. Table S3. Table S4. Table S5. Table S6. Table S7. Table S8. [file EMI4-17-e70081-s001.docx]

**SUPPLEMENTARY DATA**

**Table S1. *Experimental data for test work performed at pH2 for parameters pH, sulfide (S^2-^), sulfate (SO_4_^2-^), and chemical oxygen demand (COD) concentrations.***

| pH2 | pH | | | | | | |
| --- | --- | --- | --- | --- | --- | --- | --- |
| Day | **Blk** | **AMD** | **MW** | **RS** | **CD** | **MUD** | **RM** |
| 1 | 2.09 | 2.07 | 2.45 | 2.16 | 2.03 | 2.24 | 2.23 |
| 4 | 2.07 | 2.03 | 2.61 | 2.09 | 1.98 | 2.29 | 2.26 |
| 7 | 2.32 | 2.34 | 2.88 | 2.13 | 2.01 | 2.44 | 2.33 |
| 14 | 2.13 | 2.14 | 2.70 | 2.08 | 1.95 | 2.51 | 2.3 |
| 21 | 2.08 | 2.12 | 2.67 | 1.98 | 1.83 | 2.44 | 2.21 |
| 28 | 2.11 | 2.14 | 2.69 | 2.17 | 2.04 | 2.72 | 2.46 |
| 35 | 2.08 | 2.12 | 2.70 | 2.06 | 1.93 | 2.67 | 2.36 |
| 42 | 1.98 | 2.01 | 2.61 |  |  |  |  |
| 49 | 1.98 | 2.01 | 2.64 |  |  |  |  |
| 57 |  |  |  |  |  |  |  |
| pH2 | **COD *(mg/L)*** | | | | | | |
| Day | **Blk** | **AMD** | **MW** | **RS** | **CD** | **MUD** | **RM** |
| 1 | 2475 | 2570 | 2875 | 3025 | 3170 | 2625 | 2735 |
| 4 |  |  |  |  |  |  |  |
| 7 | 2845 | 2619 | 5204 | 2565 | 3575 | 2495 | 3910 |
| 14 | 2790 | 2720 | 4540 | 2530 | 2885 | 2740 | 2240 |
| 21 | 2820 | 2525 | 4655 | 2400 | 3330 | 2785 | 1815 |
| 28 | 2680 | 2225 | 4285 | 2415 | 3015 | 2825 | 1975 |
| 35 | 4265 | 2355 | 4600 | 2410 | 3125 | 3565 | 1720 |
| 42 |  |  |  |  |  |  |  |
| 49 | 1775 | 1605 | 3865 |  |  |  |  |
| 57 |  |  |  |  |  |  |  |
| pH2 | **SO_4_^2-^ *(mg/L)*** | | | | | | |
| Day | **Blk** | **AMD** | **MW** | **RS** | **CD** | **MUD** | **RM** |
| 1 | 2086 | 3156 | 2003 | 2143 | 2048 | 2796 | 2204 |
| 4 |  |  |  |  |  |  |  |
| 7 |  |  |  | 1804 | 1839 | 2152 | 2111 |
| 14 |  |  |  | 2017 | 2010 | 2540 | 2363 |
| 21 |  |  |  | 2094 | 2116 | 2698 | 2578 |
| 28 | 852 | 2930 | 1913 | 1982 | 2017 | 2596 | 2448 |
| 35 |  |  |  | 2131 | 2113 | 2778 | 2505 |
| 42 | 1856 | 2674 | 1745 |  |  |  |  |
| 49 | 2123 | 3227 | 2075 |  |  |  |  |
| 57 |  |  |  |  |  |  |  |
| pH2 | **S^2-^*(mg/L)*** | | | | | | |
| Day | **Blk** | **AMD** | **MW** | **RS** | **CD** | **MUD** | **RM** |
| 1 | 0.000 | 0.000 | 0.020 | 0.014 | 0.021 | 0.009 | 0.016 |
| 4 | 0.002 | 0.009 | 0.054 |  | 0.033 | 0.036 | 0.025 |
| 7 | 0.035 | 0.056 | 0.006 | 0.008 | 0.027 | 0.004 | 0.000 |
| 14 | 0.009 | 0.001 | 0.012 | 0.009 | 0.002 | 0.056 | 0.0094 |
| 21 | 0.036 | 0.030 | 0.018 | 0.014 | 0.026 | 0.005 | 0.006 |
| 28 | 0.040 | 0.022 | 0.090 | 0.001 | 0.031 | 0.008 | 0.001 |
| 35 | 0.009 | 0.000 | 0.015 | 0.286 | 0.038 | 0.023 | 0.036 |
| 42 | 0.023 | 0.005 | 0.017 |  |  |  |  |
| 49 | 0.029 | 0.020 | 0.040 |  |  |  |  |
| 57 |  |  |  |  |  |  |  |

**Table S2. *Experimental data for test work performed at pH4 for parameters pH, sulfide (S^2-^), sulfate (SO_4_^2-^), and chemical oxygen demand (COD) concentrations.***

| pH4 | pH | | | | | | |
| --- | --- | --- | --- | --- | --- | --- | --- |
| Day | **Blk** | **AMD** | **MW** | **RS** | **CD** | **MUD** | **RM** |
| 1 | 3.98 | 2.87 | 4.07 | 5.11 | 4.41 | 6.27 | 4.62 |
| 4 | 3.94 | 2.95 | 4.9 | 4.44 | 4.25 | 5.50 | 4.68 |
| 7 | 4.09 | 3.29 | 5.12 | 5.35 | 4.43 | 5.98 | 5.06 |
| 14 | 3.92 | 3.18 | 4.61 | 6.49 | 4.50 | 7.04 | 6.17 |
| 21 | 3.91 | 3.18 | 4.65 | 7.07 | 4.47 | 7.27 | 6.50 |
| 28 | 3.87 | 3.29 | 4.70 | 7.24 | 4.61 | 7.67 | 7.27 |
| 35 | 3.89 | 3.33 | 4.79 | 7.49 | 4.55 | 7.72 | 6.93 |
| 42 | 3.75 | 3.30 | 4.85 |  |  |  |  |
| 49 | 3.80 | 3.32 | 5.18 |  |  |  | 7.10 |
| 57 |  |  |  | 7.8 |  | 8.3 | 7.40 |
| pH4 | **COD *(mg/L)*** | | | | | | |
| Day | **Blk** | **AMD** | **MW** | **RS** | **CD** | **MUD** | **RM** |
| 1 | 2650 | 2860 | 3125 | 2765 | 3555 | 2635 | 2765 |
| 4 |  |  |  |  |  |  |  |
| 7 | 4419 | 2869 | 4169 | 2125 | 2860 | 3555 | 2165 |
| 14 | 2410 | 2740 | 4545 | 935 | 2835 | 1735 | 2195 |
| 21 | 2205 | 2575 | 4745 | 840 | 3145 | 950 | 1780 |
| 28 | 2015 | 1990 | 4335 | 1360 | 3215 | 755 | 1665 |
| 35 | 2160 | 2025 | 4040 | 770 | 3050 | 725 | 1740 |
| 42 |  |  |  |  |  |  |  |
| 49 | 1670 | 1710 | 3980 |  |  |  |  |
| 57 |  |  |  | 705 |  | 700 | 1705 |
| pH4 | **SO_4_^2-^ *(mg/L)*** | | | | | | |
| Day | **Blk** | **AMD** | **MW** | **RS** | **CD** | **MUD** | **RM** |
| 1 | 1983 | 3009 | 1984 | 2035 | 2048 | 2243 | 2210 |
| 4 |  |  |  |  |  |  |  |
| 7 |  |  |  | 1722 | 1842 | 2129 | 2043 |
| 14 |  |  |  | 1905 | 1910 | 2364 | 2024 |
| 21 |  |  |  | 1922 | 2036 | 2191 | 1808 |
| 28 | 1809 | 3009 | 1899 | 1887 | 1877 | 1979 | 1447 |
| 35 |  |  |  | 1928 | 2050 | 2173 | 1330 |
| 42 | 1777 | 2774 | 1765 |  |  |  |  |
| 49 | 2026 | 3099 | 2114 |  |  |  | 1326 |
| 57 |  |  |  | 1844 |  | 2108 | 1335 |
| pH4 | **S^2-^ *(mg/L)*** | | | | | | |
| Day | **Blk** | **AMD** | **MW** | **RS** | **CD** | **MUD** | **RM** |
| 1 | 0.000 | 0.000 | 0.017 | 0.027 | 0.020 | 0.02 | 0.020 |
| 4 | 0.033 | 0.015 | 0.072 | 0.089 | 0.045 | 0.10 | 0.065 |
| 7 | 0.000 | 0.015 | 0.006 | 0.357 | 0.056 | 0.86 | 0.10 |
| 14 | 0.003 | 0.018 | 0.234 | 1.424 | 0.048 | 1.0 | 1.0 |
| 21 | 0.033 | 0.005 | 0.355 | 0.190 | 0.125 | 2.8 | 14 |
| 28 | 0.039 | 0.027 | 0.100 | 0.027 | 0.085 | 1.1 | 13 |
| 35 | 0.007 | 0.002 | 0.018 | 0.328 | 0.300 | 0.59 | 36 |
| 42 | 0.027 | 0.476 | 0.035 |  |  |  |  |
| 49 | 0.032 | 0.296 | 0.056 |  |  |  | 30 |
| 57 |  |  |  | 0.053 |  | 0.02 | 0.015 |

**Table S3. *Experimental data for test work performed at pH7.5 for parameters pH, sulfide (S^2-^), sulfate (SO_4_^2-^), and chemical oxygen demand (COD) concentrations.***

| pH7.5 | pH | | | | | | |
| --- | --- | --- | --- | --- | --- | --- | --- |
| Day | **Blk** | **AMD** | **MW** | **RS** | **CD** | **MUD** | **RM** |
| 1 | 7.02 | 3.4 | 6.37 | 7.07 | 6.92 | 7.10 | 6.94 |
| 4 | 6.26 | 3.47 | 5.75 | 5.62 | 5.50 | 5.88 | 5.52 |
| 7 | 5.85 | 3.72 | 6.03 | 5.97 | 5.73 | 6.22 | 5.81 |
| 14 | 5.17 | 3.71 | 5.82 | 6.21 | 6.76 | 6.68 | 6.37 |
| 21 | 5.42 | 4.50 | 6.02 | 6.24 | 6.94 | 6.99 | 6.45 |
| 28 | 5.54 | 5.35 | 6.03 | 6.58 | 7.34 | 7.10 | 6.70 |
| 35 | 5.51 | 5.50 | 5.99 | 6.59 | 7.51 | 7.28 | 6.60 |
| 42 | 5.71 | 5.44 | 6.07 |  |  |  |  |
| 49 | 5.96 | 5.74 | 6.38 | 6.75 |  | 7.77 | 6.78 |
| 57 |  |  |  | 6.62 |  | 7.64 | 7.79 |
| pH7.5 | **COD** | | | | | | |
| Day | **Blk** | **AMD** | **MW** | **RS** | **CD** | **MUD** | **RM** |
| 1 | 3065 | 2500 | 3025 | 3485 | 3510 | 2580 | 2780 |
| 4 |  |  |  |  |  |  |  |
| 7 | 2214 | 3034 | 4184 | 2300 | 2600 | 2160 | 2185 |
| 14 | 2490 | 2590 | 4455 | 2505 | 1985 | 2165 | 2250 |
| 21 | 2115 | 2665 | 4455 | 4095 | 1725 | 1815 | 1975 |
| 28 | 2105 | 2130 | 4310 | 2810 | 2385 | 1625 | 2090 |
| 35 | 2125 | 2755 | 4415 | 2315 | 1130 | 1350 | 1870 |
| 42 |  |  |  |  |  |  |  |
| 49 | 1660 | 2030 | 4095 |  |  |  |  |
| 57 |  |  |  | 2525 |  | 1115 | 2065 |
| pH7.5 | **SO_4_^2-^** | | | | | | |
| Day | **Blk** | **AMD** | **MW** | **RS** | **CD** | **MUD** | **RM** |
| 1 | 2026 | 2983 | 1964 | 1875 | 2064 | 2058 | 2063 |
| 4 |  |  |  |  |  |  |  |
| 7 |  |  |  | 1660 | 1761 | 2199 | 2034 |
| 14 |  |  |  | 1721 | 1889 | 2203 | 1949 |
| 21 |  |  |  | 1564 | 1906 | 2090 | 1723 |
| 28 | 1890 | 2891 | 1948 | 1112 | 1875 | 1938 | 1470 |
| 35 |  |  |  | 1087 | 2078 | 1860 | 1435 |
| 42 | 1734 | 2719 | 1829 |  |  |  |  |
| 49 | 2102 | 3076 | 2120 | 1289 |  | 1430 | 1418 |
| 57 |  |  |  | 1053 |  | 1288 | 1482 |
| pH7.5 | **S^2-^** | | | | | | |
| Day | **Blk** | **AMD** | **MW** | **RS** | **CD** | **MUD** | **RM** |
| 1 | 0.000 | 0.000 | 0.016 | 0.022 | 0.046 | 0.022 | 0.028 |
| 4 | 0.010 | 0.000 | 0.067 | 0.79 | 0.071 | 0.062 | 0.048 |
| 7 | 0.025 | 0.003 | 0.000 | 6.5 | 0.099 | 0.99 | 2.4 |
| 14 | 0.028 | 0.009 | 0.280 | 28 | 0.363 | 1.20 | 15 |
| 21 | 0.030 | 0.069 | 0.178 | 50 | 0.263 | 8.5 | 26 |
| 28 | 0.059 | 0.053 | 0.178 | 89 | 0.405 | 3.5 | 99 |
| 35 | 0.018 | 0.011 | 0.069 | 108 | 0.113 | 1.9 | 58 |
| 42 | 0.026 | 0.022 | 0.120 | 91 |  |  | 52 |
| 49 | 0.044 | 0.068 | 0.125 | 114 |  | 35 | 77 |
| 57 |  |  |  | 99 |  | 25 | 61 |

**Table S4. pH - *Quality Control samples analysed with each sample batch with averages and percentage error***

| pH | Day 1 | Day 4 | Day 7 | Day 14 | Day 21 | Day 28 | Day 35 | Day 42 | Day 49 | Day 57 |
| --- | --- | --- | --- | --- | --- | --- | --- | --- | --- | --- |
| Control pH 7 | 7.02 | 7.01 | 6.98 | 7.01 | 6.99 | 6.99 | 6.98 | 6.98 | 7.01 | – |
| Control pH 7 | 6.98 | 7.02 | 7.00 | 7.03 | 6.98 | 6.96 | 6.99 | – | 6.99 | 7.03 |
| Average | 7.00 | 7.02 | 6.99 | 7.02 | 6.99 | 6.98 | 6.99 | 6.98 | 7.00 | 7.03 |
| % Error | 0.00 | 0.21 | -0.14 | 0.29 | -0.21 | -0.36 | -0.21 | -0.29 | 0.00 | 0.43 |
| Control pH 4 | 4.02 | 4.01 | 3.97 | 3.97 | 3.99 | 4.03 | 3.98 | – | – | – |
| Control pH 4 | 4.04 | 4.06 | 4.01 | 3.98 | 3.98 | 4.04 | 3.99 | 3.99 | 3.97 | 3.99 |
| Average | 4.03 | 4.04 | 3.99 | 3.98 | 3.99 | 4.04 | 3.99 | 3.99 | 3.97 | 3.99 |
| % Error | 0.75 | 0.88 | -0.25 | -0.62 | -0.37 | 0.88 | -0.37 | -0.25 | -0.75 | -0.25 |

**Table S5. COD - *Quality Control samples analysed with each sample batch with averages and percentage error***

| COD | Day 1 | Day 4 | Day 7 | Day 14 | Day 21 | Day 28 | Day 35 | Day 42 | Day 49 | Day 57 |
| --- | --- | --- | --- | --- | --- | --- | --- | --- | --- | --- |
| 1000 mg/L - control | 1010 | – | 840 | 1075 | 1140 | 1090 | 1090 | – | 1075 | – |
| 1000 mg/L - control | 1090 | – | – | – | – | – | – | – | – | – |
| Average | 1050 | – | 840 | 1075 | 1140 | 1090 | 1090 | – | 1075 | – |
| % Error | 1.00 | – | -16.00 | 7.50 | 14.00 | 9.00 | 9.00 | – | 7.50 | – |

**Table S6. Sulfide - *Quality Control samples analysed with each sample batch with averages and percentage error***

| Sulfide | Day 1 | Day 4 | Day 7 | Day 14 | Day 21 | Day 28 | Day 35 | Day 42 | Day 49 | Day 57 |
| --- | --- | --- | --- | --- | --- | --- | --- | --- | --- | --- |
| 0.5 mg/L control | 0.53 | 0.51 | 0.42 | 0.43 | 0.47 | – | 0.45 | 0.409 | – | 0.45 |
| % Error | 6.84 | 1.38 | -16.83 | -14.44 | -6.42 | – | -10.28 | -18.26 | – | -10.09 |
| 1.0 mg/L control | 1.07 | 1.04 | 0.98 | 1.01 | 0.98 | 0.73 | 1.03 | 0.986 | 1.00 | 1.02 |
| % Error | 6.73 | 3.80 | -1.60 | 0.97 | -1.73 | -27.30 | 3.41 | -1.43 | 0.27 | 2.02 |

**Table S7. Sulfate - *Quality Control samples analysed with each sample batch with averages and percentage error***

| Sulfate | Day 1 | Day 4 | Day 7 | Day 14 | Day 21 | Day 28 | Day 35 | Day 42 | Day 49 | Day 57 |
| --- | --- | --- | --- | --- | --- | --- | --- | --- | --- | --- |
| 10 mg/L - control | – | – | – | 10.1 | – | 10.0 | 10.0 | 10.8 | 9.06 | 9.68 |
| 10 mg/L - control | – | – | – | 9.89 | – | 10.1 | – | – | 10.4 | – |
| Average | – | – | – | 10.0 | – | 10.1 | 10.0 | 10.8 | 9.74 | 9.68 |
| % Error | – | – | – | 0.06 | – | 0.54 | 0.32 | 7.75 | -2.58 | -3.20 |
| 20 mg/L - control | 21.09 | – | – | 20.6 | 20.6 | 20.9 | 20.9 | 21.4 | 21.3 | – |
| % Error | 5.44 | – | – | 2.75 | 2.75 | 4.44 | 4.29 | 7.13 | 6.25 | – |
| 25 mg/L - control | – | – | 24.1 | – | – | – | – | 24.1 | – | – |
| 25 mg/L - control | – | – | 24.3 | – | – | – | – | 24.3 | – | – |
| Average | – | – | 24.2 | – | – | – | – | 24.2 | – | – |
| % Error | – | – | -3.10 | – | – | – | – | -3.10 | – | – |

**Table S8. Sulfate – *Sample replicate analysis with averages and standard deviations***

| Sulfate - replicate analysis | Day 1 | Day 4 | Day 7 | Day 14 | Day 21 | Day 28 | Day 35 | Day 42 | Day 49 | Day 57 |
| --- | --- | --- | --- | --- | --- | --- | --- | --- | --- | --- |
| AMD pH 2 | 3156 | – | – | – | – | – | – | – | – | – |
| AMD pH 2 | 3143 | – | – | – | – | – | – | – | – | – |
| Average | 3149 | – | – | – | – | – | – | – | – | – |
| Std dev | **9.12** | – | – | – | – | – | – | – | – | – |
| Blk pH 4 | 1983 | – | – | – | – | – | – | – | – | – |
| Blk pH 4 | 1990 | – | – | – | – | – | – | – | – | – |
| Average | 1987 | – | – | – | – | – | – | – | – | – |
| Std dev | 4.77 | – | – | – | – | – | – | – | – | – |
| Sewage 7.5 | – | – | – | 1721 | – | – | 1087 | – | – | – |
| Sewage 7.5 | – | – | – | 1797 | – | – | 1154 | – | – | – |
| Average | – | – | – | 1759 | – | – | 1121 | – | – | – |
| Std dev | – | – | – | 53.54 | – | – | 47.38 | – | – | – |
| Reed Mud 7.5 | – | – | – | – | 1470 | – | – | – | 1326 | 1335 |
| Reed Mud 7.5 | – | – | – | – | 1458 | – | – | – | 1418 | 1482 |
| Average | – | – | – | – | 1464 | – | – | – | 1372 | 1408 |
| Std dev | – | – | – | – | 8.32 | – | – | – | 65.47 | 104.13 |
